# Final Exon Frameshift Biallelic *PTPN23* Variants Are Associated with Microcephalic Complex Hereditary Spastic Paraplegia

**DOI:** 10.3390/brainsci11050614

**Published:** 2021-05-11

**Authors:** Reham Khalaf-Nazzal, James Fasham, Nishanka Ubeyratna, David J. Evans, Joseph S. Leslie, Thomas T. Warner, Fida’ Al-Hijawi, Shurouq Alshaer, Wisam Baker, Peter D. Turnpenny, Emma L. Baple, Andrew H. Crosby

**Affiliations:** 1 Biomedical Sciences Department, Faculty of Medicine, Arab American University of Palestine, Jenin P227, Palestine; 2 College of Medicine and Health, RILD Wellcome Wolfson Centre, University of Exeter, Royal Devon & Exeter NHS Foundation Trust, Barrack Road, Exeter EX2 5DW, UK; j.fasham@exeter.ac.uk (J.F.); nu213@exeter.ac.uk (N.U.); j.leslie@exeter.ac.uk (J.S.L.); peter.turnpenny@nhs.net (P.D.T.); 3 Peninsula Clinical Genetics Service, Royal Devon & Exeter Hospital (Heavitree), Gladstone Road, Exeter EX1 2ED, UK; 4 Exeter Genomics Laboratory, Royal Devon & Exeter NHS Foundation Trust, Exeter EX2 5DW, UK; david.evans34@nhs.net; 5 Reta Lila Weston Institute, UCL Queen Square Institute of Neurology, 1 Wakefield Street, London WC1N 1PJ, UK; t.warner@ucl.ac.uk; 6 Paediatrics’ Community Outpatient Clinics, Palestinian Ministry of Health, Jenin P200, Palestine; Hajfida7@gmail.com; 7 Faculty of Graduate Studies, Arab American University, Ramallah P622, Palestine; shoroqalshaer362@gmail.com; 8 Paediatrics Department, Dr. Khalil Suleiman Government Hospital, Jenin P200, Palestine; wisambaker30@yahoo.com

**Keywords:** HSP, hereditary spastic paraplegia, *PTPN23*, protein tyrosine phosphatase, ESCRT

## Abstract

The hereditary spastic paraplegias (HSPs) are a large clinically heterogeneous group of genetic disorders classified as ‘pure’ when the cardinal feature of progressive lower limb spasticity and weakness occurs in isolation and ‘complex’ when associated with other clinical signs. Here, we identify a homozygous frameshift alteration occurring in the last coding exon of the protein tyrosine phosphatase type 23 (*PTPN23*) gene in an extended Palestinian family associated with autosomal recessive complex HSP. *PTPN23* encodes a catalytically inert non-receptor protein tyrosine phosphatase that has been proposed to interact with the endosomal sorting complex required for transport (ESCRT) complex, involved in the sorting of ubiquitinated cargos for fusion with lysosomes. In view of our data, we reviewed previously published candidate pathogenic *PTPN23* variants to clarify clinical outcomes associated with pathogenic gene variants. This determined that a number of previously proposed candidate *PTPN23* alterations are likely benign and revealed that pathogenic biallelic *PTPN23* alterations cause a varied clinical spectrum comprising of complex HSP associated with microcephaly, which may occur without intellectual impairment or involve more severe neurological disease. Together, these findings highlight the importance of the inclusion of the *PTPN23* gene on HSP gene testing panels globally.

## 1. Introduction

The hereditary spastic paraplegias (HSPs) are a heterogeneous group of monogenic neurodegenerative diseases characterised by progressive spasticity of the lower limbs, with a pooled global prevalence of 1.8/100,000 [1]. In clinical practice, HSPs are subclassified into either (i) uncomplicated (or pure) when neurologic impairment is limited to progressive lower-extremity spastic and weakness, hypertonic urinary bladder disturbance, and mild diminution of lower-extremity vibration sensation or (ii) complicated (or complex) when these features are accompanied by other neurological or non-neurological signs [2]. Over recent years, advancements in our understanding of the genetic architecture of HSP have led to it being recognised as one of the most genetically heterogeneous of inherited disorders, with pathogenic sequence alterations in affected families identified in at least 72 genes in molecules associated with a plethora of cellular roles [3]. In addition, many other genetic disorders have also been described in which spasticity is a key diagnostic feature, underscoring the immense clinical, genetic, and molecular complexities of this clinical presentation [4,5]. 

*PTPN23* encodes the ubiquitously expressed, non-receptor protein tyrosine phosphatase (PTPN) type 23, also known as the histidine-rich (HIS)-domain protein tyrosine phosphatase (HDPTP) [6]. PTPNs have a well-defined function in cellular signal transduction by regulating tyrosine residue phosphorylation [7,8]. The specific cellular roles of *PTPN23* include interactions with mitogen-activated protein kinase signalling (MAPK) pathways [9], ciliogenesis [10], and regulation of splicing through regulation of survival of motor neurone (SMN) [11]. PTPN23 has also been shown to interact with the endosomal sorting complex required for transport (ESCRT) involved in the sorting of ubiquitinated cargos into multivesicular bodies (MVBs) for fusion with lysosomes and cargo protein degradation [12]. Here we present our genetic and clinical findings of *PTPN23*-related complex HSP identified in a Palestinian community, alongside a review of recently published candidate *PTPN23* sequence alterations which together define biallelic *PTPN23* sequence alterations as a cause of complex HSP associated with microcephaly.

## 2. Materials and Methods

### Genetic Studies

Blood samples were obtained with informed consent (Ethical Approval; the Palestinian Health Research Council PHRC/HC/518/19) for DNA extraction using standard procedures. Whole exome sequencing (WES) was performed in-house, using the Twist Human Core Exome capture on an Illumina NextSeq500 sequencer. Reads were aligned to the human genome reference sequence (hg19) using BWA-MEM (v0.7.17), mate pairs were fixed and duplicates removed using Picard (v2.15), InDel realignment and base quality recalibration were performed using GATK (v3.7.0), SNVs and InDels were detected using GATK HaplotypeCaller and annotated using Alamut batch (v1.10). Read depth was determined for the whole exome using GATK DepthOfCoverage, conforming to GATK Best Practices. Copy number variants (CNVs) were detected using SavvyCNV [13]. Variants were then filtered on call quality, gnomAD allele frequency, presence in databases of pathogenic variants, and inheritance pattern. Orthogonal validation of the *PTPN23* variant was undertaken by dideoxy sequencing. 

## 3. Results

### 3.1. Clinical Findings

**Figure 1:**
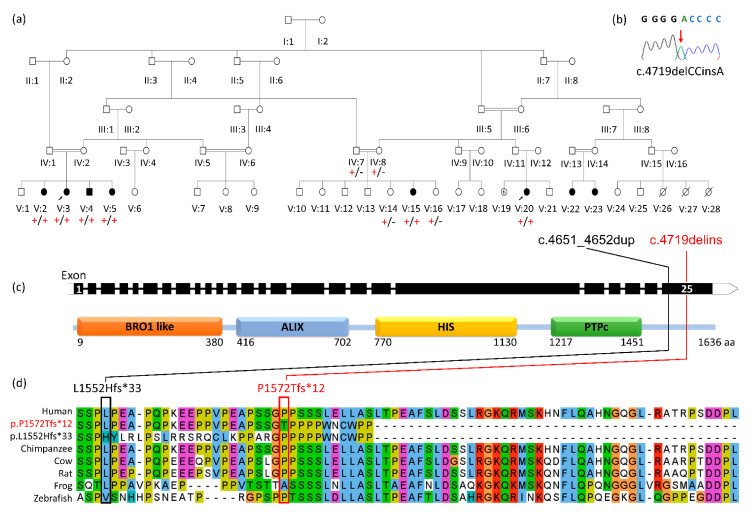
Truncating *PTPN23* gene variants that escape nonsense-mediated decay are associated with complex spastic paraplegia: (**a**) simplified pedigree of the Arab Palestinian family investigated, demonstrating segregation of the *PTPN23* variant [‘+’: NM_015466.3:c.4719delins p.(Pro1572Thrfs*12), ‘-’: wild type]. (**b**) Electropherogram revealing the DNA sequence of the *PTPN23* NM_015466.3:c.4719delins variant in homozygous state in an affected individual (Individual V:4). (**c**) A simplified gene diagram showing exon-intron organisation of *PTPN23* (NM_015466.3) and the corresponding domain architecture of the PTPN23 protein. The position of the NM_015466.3:c.4719delins p. Pro1572Thrfs*12 variant is shown [red line] in relation to the NM_015466.3:c.4651_4652dup; p.(Leu1552Hisfs*33) [14] [black line]. (**d**). PTPN23 protein alignment of human wild type and five species orthologues, alongside the predicted outcomes of the p.(Pro1572Thrfs*12) and p.(Leu1552Hisfs*33) variants.

In the current study, eight individuals affected by a microcephalic form of complex HSP were identified from a single extended pedigree. The family entails four interrelated nuclear Arab Palestinian families from the same community living in the West Bank (Figure 1), each likely sharing distant common ancestors. Of these, six individuals aged between 10 and 25 years old were available for genetic studies and detailed clinical phenotyping (Individuals V:2, V:3, V:4, V:5, V15, and V20 (Table 1)). 

All affected individuals displayed progressive, lower-limb spasticity with associated hyperreflexia, upgoing plantar responses, and muscle weakness. This resulted in a narrow-based gait with evidence of proximal spasticity, bilateral foot drop, and excessive lumbar lordosis in Individual V:4 (Video 1). Symptoms consistent with paraesthesia in the distal lower limbs were also described, but the sensory examination was unremarkable. Lower limb nerve conduction studies were performed in Individual V:20 and were normal. Upper limb reflexes, motor function, and sensation were all unaffected, and there was no clinical evidence of bulbar involvement. Although all affected individuals were microcephalic (−2.5 to −3.8 standard deviations [SDs]), there was a variable degree of intellectual impairment from mild (able to read and write) to normal (two individuals were in tertiary education, V:4, V:20), and none were affected by seizures. Early motor milestones were not delayed (all individuals walking by 14 months), although there were subtle signs of neurological impairment in the first decade with toe walking indicative of spasticity, being the first sign in most and requiring surgery in Individual V:4. Typically, by age 6–7 years, affected individuals were noted to have developed an unsteady gait with frequent falling which worsened progressively. Around the same age, affected individuals developed nystagmus. MRI neuroimaging was performed in Individual V:3 and V:4 and demonstrated no structural pathology. 

### 3.2. Genetic Findings

Assuming that an autosomal recessive founder variant was responsible for the condition, WES was performed on DNA from Individuals V:3 and V:20 to identify candidate sequence variants common to both. Plausible compound heterozygous and structural variants located genome wide were also considered, but none were identified that cosegregated with the condition. Filtering identified a complex deletion/insertion within the coding region of the *PTPN23* gene [Chr3(GRCh38):g.47412993delCCinsA NM_015466.3:c.4719delins p. Pro1572Thrfs*12] (Figure 1b) predicted to cause a frameshift in the last exon and thus would be expected to escape nonsense-mediated decay, resulting in a polypeptide truncated by 64 amino acids (1572, compared to the wild type 1636). The *PTPN23* variant was confirmed using dideoxy sequencing and found to cosegregate among all family members as expected for an autosomal recessive cause of the disease (Figure 1a). The variant is listed in heterozygous state in only one African/African American individual in gnomAD v2.1.1, was not reported in homozygous state, is absent from gnomAD v3.1, and has not been previously reported in ClinVar or HGMDPro. This variant is located within a likely autozygous ~26Mb region of homozygosity shared by both affected individuals, also containing one other rare missense variant in the gene BSN [Chr3(GRCh38):g. 49652264C>T NM_003458.3:c.2708C>T p.Thr903Met]. The variant was predicted damaging by both SiFT and PolyPhen2, but the gene has no known association with human disease.

## 4. Discussion

Here, we present our clinical and genetic findings of a complex form of hereditary spastic paraplegia associated with biallelic *PTPN23* variants in an extended Palestinian kinship of eight affected individuals, six of whom were available for investigation. The c.4719delins variant identified in this study resides within the last exon of the *PTPN23* gene and therefore is predicted to escape nonsense-mediated decay. This alteration may thus result in the production of a modestly truncated PTPN23 polypeptide product with an altered C-terminus (Figure 1d), although molecular studies are required to confirm this and the degree to which the mutant molecule produced retains functionality. 

Previously, four individual case reports have identified *PTPN23* gene alterations as a candidate cause of neurological disease in four unrelated individuals with severe epilepsy and neurodevelopmental delay, sometimes classified as developmental and epileptic encephalopathy (DEE; [ILAE classification]) (Table S1) [15,16,17,18]. Subsequently, a further study identified biallelic candidate *PTPN23* gene variants in seven additional individuals affected by a variable degree of neurodevelopmental impairment, with structural brain abnormalities described in some patients [14]. However, current publicly available gnomAD allele frequency data do not support pathogenicity for the *PTPN23* gene variants identified in two of the individuals in these studies (Table S2) [14]. Additionally, the clinical features described in a further three of these cases are relatively non-specific and diverge considerably from the primarily neurological outcomes associated with the originally described pathogenic *PTPN23* variants (Patients 1–3, Table S3). 

In the extended Palestinian family investigated here, all affected individuals displayed lower limb weakness, muscle wasting, hyperreflexia, and upgoing plantar reflexes, with clawed toes being noted in older affected individuals. The gait displays features of both spasticity and foot drop with distal weakness and wasting. The normal sensory examination and nerve conduction studies are in keeping with a motor neuronopathy, suggesting pathology with combined upper and lower neuron involvement similar to that seen in SPG17 (Silver syndrome) [19]. All affected individuals were also microcephalic, and the majority (but notably not all) had mild intellectual impairment. These clinical features align closely with those in a previously reported case (Table 1: Bend et al. Patient 6) in whom a distinct C-terminal frameshift [NM_015466.3:c.4651_4652dup; p.(Leu1552Hisfs*33)] variant was identified, which closely mirrors the c.4719delins p.(Pro1572Thrfs*12) Palestinian *PTPN23* alteration and is predicted to result in truncation of the protein to exactly the same length (1572/1636 amino acids). This individual also has microcephaly and developmental impairment, spastic diplegia, and contractures at eleven years of age, in the absence of seizures. While molecular studies are required to investigate this further, the consistent clinical outcomes defined in all of these individuals (Table 1) indicates that *PTPN23* last exon C-terminal frameshift gene mutations that occur subsequent (C-terminal) to the tyrosine–protein phosphatase domain, may give rise to a polypeptide product which retains partial functionality. The C-terminal disrupted by these mutations is proline rich (Figure 1d); although no specific function for this region has yet been identified, another such proline rich PTPN23 region (the HIS domain) has been shown to be of functional importance for protein–protein interaction with growth factor receptor-bound protein 2 (Grb2) family of proteins [20]. Although the C-terminal proline-rich domain was shown not to modulate this interaction, it is possible that it is involved in other protein–protein interactions. 

While overlapping, a more severe neurological phenotype involving seizures, microcephaly, progressive spasticity, brain atrophy, and hypomyelination of white matter appears to be associated with *PTPN23* missense variants located within the BRO1-like [16], tyrosine–protein phosphatase [15], and ALIX domains [17,18] (Table S1, Figure S4). This indicates a more deleterious outcome of variants within these functionally important regions, associated with more severe clinical signs. The importance of the BRO domain and its paired ALIX domain is unsurprising since it is conserved among many *PTPN23* orthologues and also observed in human programmed cell death six-interacting protein (PDCD6IP), which also interacts with ESCRT complexes. The ALIX domain in particular has been identified to have an important role in interacting with and recruiting such complexes [21]. Although the tyrosine–protein phosphatase domain has previously been shown to be catalytically inert [11], it seems likely to have acquired other as yet unknown functional roles. As the BRO/PTP/ALIX missense alterations identified by Sowada et al. [17] and Smigiel et al. [18] (Table S1) occurred in conjunction with a loss of function alteration, any polypeptide produced would likely derive from the missense-harbouring allele only, assuming degradation of the loss of function allele. As such, the similarly severe neurological disease may thus also be expected in individuals found to be homozygous/compound heterozygous for missense alterations in these regions.

In humans, 17 PTPNs have been identified [22], each with their individual expression patterns and characterised by different regulatory sequences that flank the catalytic domain and modulate activity or control substrate specificity [8], most of which do not currently have an associated human disorder resulting from their mutation. A notable exception is *PTPN11* involved in the Ras/MAPK signal transduction pathway, the most common gene associated with the autosomal dominant developmental disorder Noonan syndrome [23,24]. *PTPN23* encodes a catalytically inactive non-receptor protein tyrosine phosphatase [11] with an ESCRT-I-related role in endocytic sorting of ubiquitinated cargos into multivesicular bodies [12], as well as likely roles in ciliogenesis [10], regulation of the survival motor neuron (SMN) complex function in the assembly of splicing factors [6] and as a negative regulator of Ras-mediated mitogenic activity [9]. Four PTPN23 polypeptide functional domains have been identified, including an ALIX domain believed to have an important ESCRT complex role [21]. ESCRT complexes are concentrated at pre- and postsynaptic sites and likely have a role in synaptic vesicle recycling, degradation, and growth. Previous studies support the hypothesis that dysfunction of the ESCRT complex may cause neurodegeneration presenting as HSP including variants in ubiquitin associated protein 1 (UBAP1), a component of the ESCRT-I complex, in which variants have recently been associated with autosomal dominant pure HSP (SPG80) with childhood onset [25]. In addition, the most common cause of HSP involving variants in spastin (SPG4) has a well-documented role in interacting with the ESCRT-III complex-associated endosomal protein CHMP1B [26,27] via an MIT domain (contained within microtubule interacting and trafficking molecules) [28], also present in spartin (SPG20) [29], another HSP-associated molecule [30,31]. This provides a potential mechanism to explain the important role of PTPN23 function in the long-term health of motor neurones and the role of PTPN23 mutation in complex HSP4.

## 5. Conclusions

Taken together, our findings define pathogenic biallelic *PTPN23* variants as a cause of a variable clinical spectrum of neurological disease comprising of complex HSP associated with microcephaly, which may occur without intellectual impairment, or involve more severe neurological disease. Given that intellectual impairment may be absent in this clinically variable condition, it is important that *PTPN23* be considered for inclusion on spastic paraplegia gene testing-diagnostic panels internationally. 

## Figures and Tables

**Table 1:** A comparison of clinical findings of affected individuals homozygous for final exon frameshift *PTPN23* gene variants.

Reference	V:2	V:3	V:4	V:5	V:15	V:20	Bend et al. Patient 6. [14]
Genotype	+/+	+/+	+/+	+/+	+/+	+/+	p.(Leu1552Hisfs*33) /p.(Leu1552Hisfs*33)
Sex, Age last seen	F, 17y10m	F, 14y2m	M, 22y4m	F, 10y1m	F, 16y11m	F, 25y1m	F, 11y
Age of onset	6y	6y	4-5y	6y	7y	7y	*NK*
OFC (cm) [SD^1^]	50.5 [−3.8]	50.8 [−3.1]	*NK*	49.2 [−3.6]	52 [−2.5]	50.8 [−3.4]	microcephaly
Height (cm) [SD^1^]	152 [−1.9]	143 [−2.7]	169 [−1.3]	134.5 [−0.7]	152 [−1.9]	152 [−2.0]	*NK*
Weight (kg) [SD^1^]	66 [+0.9]	59 [+0.9]	66 [−0.6]	*NK*	*NK*	55 [−0.4]	*NK*
Dev. impairment	✓mild	✓mild	✘ university	✓mild	✓mild	✘ university	✓no speech
Toe walking	✘	✓	✓	✓	✓	✓	*NK*
Speech delay	✘	✘	✘	✘	✘	✘	*NK*
Upper limb neurology	normal	normal	normal	normal	normal	normal	*NK*
Lower limb spasticity	✓	✓	✓	✓	✓	✓	✓
Lower limb DTRs	+++	+++	+++	+++	+++	+++	*NK*
Babinski reflex	↑	↑	↑	↑	↑	↑	*NK*
Hypo/paraesthesia	✘	✓episodic	✓episodic	✓episodic	✓	✓	*NK*
Light touch sensation	normal	normal	normal	normal	normal	normal	*NK*
Pain sensation	normal	normal	normal	normal	normal	normal	*NK*
Seizures	✘	✘	✘	✘	✘	✘	✘ normal EEG
Bulbar features	✘	✘	✘	✘	✘	✘	*NK*
Sphincter dysfunction	✘	✘	✘	✘	✘	✘	*NK*
Optic atrophy	*NK*	*NK*	*NK*	*NK*	*NK*	*NK*	✓& strabismus
Horizontal nystagmus	✓	✓	✓	✓	✘	✓	*NK*
MRI brain	*NP*	normal	normal	*NP*	*NP*	*NP*	enlarged lateral ventricle, delayed myelination
Other					DDH, dysphagia		constipation

Note: (+): NM_015466.3:c.4719delins p.(Pro1572Thrfs*12), (↑): upgoing, (✓): indicates presence of a feature in an affected subject, (✘): indicates absence of a feature in an affected subject, (+++): exaggerated reflexes, cm: centimetres, DDH: developmental dysplasia of the hip, Dev: Developmental, DTRs: deep tendon reflexes, EEG: electroencephalogram, F: female, m: months, M: male, MRI: magnetic resonance imaging, NK: not known, NP: not performed: OFC: occipitofrontal circumference, SD: standard deviations, y: years. ^1^ Height, weight, BMI, and OFC Z-scores were calculated using LMS growth, a Microsoft Excel add-in to access growth references based on the LMS method (https://www.healthforallchildren.com/lmsgrowth/ accessed 07 May 2021).

## Data Availability

Data regarding the identified variant and associated clinical features have been deposited in ClinVar (SUB9463082).

## Supplementary Materials

The following are available online at https://www.mdpi.com/article/10.3390/brainsci11050614/s1, Table S1: Candidate PTPN23 gene variants identified in patients with severe neurological impairment and seizures, Table S2: Candidate PTPN23 gene variants, proposed by Bend et al. 2020, Table S3: Candidate PTPN23 gene variants identified in patients with non-specific developmental impairment by Bend et al. 2020 not excluded by allele frequency data in population databases, Supplementary Figure S4: Position of previously published PTPN23 candidate missense variants and small in-frame deletions in relation to known PTPN23 protein domain architecture, Video 1: A demonstration of the gait in the PTPN23-related complex hereditary spastic paraplegia (Individual V4).

## References

[1] Ruano L., Melo C., Silva M.C., Coutinho P. (2014). The global epidemiology of hereditary ataxia and spastic paraplegia: A systematic review of prevalence studies. Neuroepidemiology.

[2] Harding A.E. (1983). Classification of the hereditary ataxias and paraplegias. Lancet.

[3] Genomics England Hereditary Spastic Paraplegia (Version 1.219) 2021. https://panelapp.genomicsengland.co.uk/panels/165/.

[4] Kara E., Tucci A., Manzoni C., Lynch D.S., Elpidorou M., Bettencourt C., Chelban V., Manole A., Hamed S.A., Haridy N.A. (2016). Genetic and phenotypic characterization of complex hereditary spastic paraplegia. Brain J. Neurol..

[5] Laurá M., Pipis M., Rossor A.M., Reilly M.M. (2019). Charcot-Marie-Tooth disease and related disorders: An evolving landscape. Curr. Opin. Neurol..

[6] Husedzinovic A., Neumann B., Reymann J., Draeger-Meurer S., Chari A., Erfle H., Fischer U., Gruss O.J. (2015). The catalytically inactive tyrosine phosphatase HD-PTP/PTPN23 is a novel regulator of SMN complex localization. Mol. Biol. Cell..

[7] Tonks N.K. (2013). Protein tyrosine phosphatases--from housekeeping enzymes to master regulators of signal transduction. FEBS J..

[8] Tonks N.K. (2006). Protein tyrosine phosphatases: From genes, to function, to disease. Nat. Rev. Mol. Cell Biol..

[9] Cao L., Zhang L., Ruiz-Lozano P., Yang Q., Chien K.R., Graham R.M., Zhou M. (1998). A novel putative protein-tyrosine phosphatase contains a BRO1-like domain and suppresses Ha-ras-mediated transformation. J. Biol. Chem..

[10] Kim J., Lee J.E., Heynen-Genel S., Suyama E., Ono K., Lee K., Ideker T., Aza-Blanc P., Gleeson J.G. (2010). Functional genomic screen for modulators of ciliogenesis and cilium length. Nature.

[11] Gingras M.C., Zhang Y.L., Kharitidi D., Barr A.J., Knapp S., Tremblay M.L., Pause A. (2009). HD-PTP is a catalytically inactive tyrosine phosphatase due to a conserved divergence in its phosphatase domain. PLoS ONE.

[12] Doyotte A., Mironov A., McKenzie E., Woodman P. (2008). The Bro1-related protein HD-PTP/PTPN23 is required for endosomal cargo sorting and multivesicular body morphogenesis. Proc. Natl. Acad. Sci. USA.

[13] Laver T.W., Franco E.D., Johnson M.B., Patel K., Ellard S., Weedon M.N., Flanagan S.E., Wakeling M.N. (2019). SavvyCNV: Genome-wide CNV calling from off-target reads. BioRxiv.

[14] Bend R., Cohen L., Carter M.T., Lyons M.J., Niyazov D., Mikati M.A., Rojas S.K., Person R.E., Si Y., Wentzensen I.M. (2020). Phenotype and mutation expansion of the PTPN23 associated disorder characterized by neurodevelopmental delay and structural brain abnormalities. Eur. J. Hum. Genet..

[15] Alazami A.M., Patel N., Shamseldin H.E., Anazi S., Al-Dosari M.S., Alzahrani F., Hijazi H., Alshammari M., Aldahmesh M.A., Salih M.A. (2015). Accelerating novel candidate gene discovery in neurogenetic disorders via whole-exome sequencing of prescreened multiplex consanguineous families. Cell Rep..

[16] Trujillano D., Bertoli-Avella A.M., Kumar Kandaswamy K., Weiss M.E., Köster J., Marais A., Paknia O., Schröder R., Garcia-Aznar J.M., Werber M. (2017). Clinical exome sequencing: Results from 2819 samples reflecting 1000 families. Eur. J. Hum. Genet..

[17] Sowada N., Hashem M.O., Yilmaz R., Hamad M., Kakar N., Thiele H., Arold S.T., Bode H., Alkuraya F.S., Borck G. (2017). Mutations of PTPN23 in developmental and epileptic encephalopathy. Hum. Genet..

[18] Smigiel R., Landsberg G., Schilling M., Rydzanicz M., Pollak A., Walczak A., Stodolak A., Stawinski P., Mierzewska H., Sasiadek M.M. (2018). Developmental epileptic encephalopathy with hypomyelination and brain atrophy associated with PTPN23 variants affecting the assembly of UsnRNPs. Eur. J. Hum. Genet..

[19] Windpassinger C., Auer-Grumbach M., Irobi J., Patel H., Petek E., Hörl G., Malli R., Reed J.A., Dierick I., Verpoorten N. (2004). Heterozygous missense mutations in BSCL2 are associated with distal hereditary motor neuropathy and Silver syndrome. Nat. Genet..

[20] Tanase C.A. (2010). Histidine domain-protein tyrosine phosphatase interacts with Grb2 and GrpL. PLoS ONE.

[21] Zhai Q., Fisher R.D., Chung H.Y., Myszka D.G., Sundquist W.I., Hill C.P. (2008). Structural and functional studies of ALIX interactions with YPX(n)L late domains of HIV-1 and EIAV. Nat. Struct. Mol. Biol..

[22] Alonso A., Sasin J., Bottini N., Friedberg I., Friedberg I., Osterman A., Godzik A., Hunter T., Dixon J., Mustelin T. (2004). Protein Tyrosine Phosphatases in the Human Genome. Cell.

[23] Tartaglia M., Mehler E.L., Goldberg R., Zampino G., Brunner H.G., Kremer H., Van der Burgt I., Crosby A.H., Ion A., Jeffery S. (2001). Mutations in PTPN11, encoding the protein tyrosine phosphatase SHP-2, cause Noonan syndrome. Nat. Genet..

[24] Shaw A.C., Kalidas K., Crosby A.H., Jeffery S., Patton M.A. (2007). The natural history of Noonan syndrome: A long-term follow-up study. Arch. Dis. Child..

[25] Farazi Fard M.A., Rebelo A.P., Buglo E., Nemati H., Dastsooz H., Gehweiler I., Reich S., Reichbauer J., Quintáns B., Ordóñez-Ugalde A. (2019). Truncating Mutations in UBAP1 Cause Hereditary Spastic Paraplegia. Am. J. Hum. Genet..

[26] Reid E., Connell J., Edwards T.L., Duley S., Brown S.E., Sanderson C.M. (2004). The hereditary spastic paraplegia protein spastin interacts with the ESCRT-III complex-associated endosomal protein CHMP1B. Hum. Mol. Genet..

[27] Connell J.W., Allison R.J., Rodger C.E., Pearson G., Zlamalova E., Reid E. (2019). ESCRT-III-associated proteins and spastin inhibit protrudin-dependent polarised membrane traffic. Cell. Mol. Life Sci..

[28] Ciccarelli F.D., Proukakis C., Patel H., Cross H., Azam S., Patton M.A., Bork P., Crosby A.H. (2003). The identification of a conserved domain in both spartin and spastin, mutated in hereditary spastic paraplegia. Genomics.

[29] Patel H., Cross H., Proukakis C., Hershberger R., Bork P., Ciccarelli F.D., Patton M.A., McKusick V.A., Crosby A.H. (2002). SPG20 is mutated in Troyer syndrome, an hereditary spastic paraplegia. Nat. Genet..

[30] Renvoisé B., Parker R.L., Yang D., Bakowska J.C., Hurley J.H., Blackstone C. (2010). SPG20 protein spartin is recruited to midbodies by ESCRT-III protein Ist1 and participates in cytokinesis. Mol. Biol. Cell..

[31] Proukakis C., Cross H., Patel H., Patton M.A., Valentine A., Crosby A.H. (2004). Troyer syndrome revisited. A clinical and radiological study of a complicated hereditary spastic paraplegia. J. Neurol..

