# Supplementary material for: Final Exon Frameshift Biallelic PTPN23 Variants Are Associated with Microcephalic Complex Hereditary Spastic Paraplegia
Source: Brain Sci. 2021 May 11;11(5):614. doi: 10.3390/brainsci11050614 (PMC8151426; doi:10.3390/brainsci11050614)
Supplement: Supplementary file 1 [file brainsci-11-00614-s001.zip › brainsci-1200361-supplementary/brainsci-1200361-supplementary.pdf]

Final exon frameshift biallelic *PTPN23* variants are associated with microcephalic complex hereditary spastic paraplegia.

Supplemental Paper

Reham Khalaf-Nazzal[1,#,*], James Fasham[2,3#], Nishanka Ubeyratna[2], David Evans[4], Joseph S Leslie[2], Thomas T Warner[5], Fida' Al-Hijawi[6], Shurouq Alshaer[7,] Wisam Baker[8], Peter D Turnpenny[2,3], Emma L Baple[2,3,$*], Andrew H Crosby[2,$,*]

1 Biomedical Sciences Department, Faculty of Medicine, Arab American University of Palestine, Jenin, Palestine; reham.nazzal@aaup.edu
2 College of Medicine and Health, RILD Wellcome Wolfson Centre, University of Exeter, Royal Devon & Exeter NHS Foundation Trust, Barrack Road, Exeter, EX2 5DW, UK; j.fasham@exeter.ac.uk; nu213@exeter.ac.uk; j.leslie@exeter.ac.uk; e.baple@exeter.ac.uk; a.h.crosby@exeter.ac.uk
3 Peninsula Clinical Genetics Service, Royal Devon & Exeter Hospital (Heavitree), Gladstone Road, Exeter, EX1 2ED, UK; J.Fasham@exeter.ac.uk; e.baple@exeter.ac.uk peter.turnpenny@nhs.net
4. Exeter Genomics Laboratory, Royal Devon & Exeter NHS Foundation Trust, Exeter, UK; david.evans34@nhs.net
5. Reta Lila Weston Institute, UCL Queen Square Institute of Neurology, 1 Wakefield Street, London WC1N 1PJ; t.warner@ucl.ac.uk
6. Paediatrics' Community Outpatient Clinics, Palestinian Ministry of Health. Jenin, Palestine; Hajfida7@gmail.com
7. Faculty of Graduate Studies, Arab American University. Ramallah, Palestine; shoroqalshaer362@gmail.com
8. Paediatrics Department, Dr. Khalil Suleiman Government hospital. Jenin, Palestine, wisambaker30@yahoo.com
# These authors contributed equally to this work
$ These authors contributed equally to this work
* Correspondence: a.h.crosby@exeter.ac.uk; e.baple@exeter.ac.uk; reham.nazzal@aaup.edu

Citation: Nazzal, R.; Fasham, J. *et al.* F. Final exon frameshift biallelic *PTPN23* variants are associated with microcephalic complex hereditary spastic paraplegia. *Brain Sci.* **2021**, *11*, x. https://doi.org/10.3390/xxxxx

Academic Editor: Firstname Lastname

Received: date
Accepted: date
Published: date

Publisher's Note: MDPI stays neutral with regard to jurisdictional claims in published maps and institutional affiliations.

Copyright: © 2021 by the authors. Submitted for possible open access publication under the terms and conditions of the Creative Commons Attribution (CC BY) license (https://creativecommons.org/licenses/by/4.0/).

Table S1. Candidate *PTPN23* gene variants identified in patients with severe neurological impairment and seizures.

**Reference**	**Alazami**[1]	**Trujillano**[2]	**Sowada**[3]	**Smigiel**[4]	**Bend[5]**<br>**Patient 4**
Genotype (NM_015466.4)	Homozygous<br>c.3995G>T: p.(Arg1332Leu)	Homozygous<br>c.904A>G p.(Met302Val)	c.1595C>T p.(Pro532Leu)<br>| c.3586C>T p.(Arg1196*)	c.1902C>G p.(Asn634Lys)<br>| c.2974delC;<br>p.(Leu992Tyrfs*168)	Homozygous<br>c.2568_2594del27;<br>p.(Val857_Pro865del)
Family history	Parents are cousins<br>Two similar deceased siblings	NK	Isolated case	Isolated case	
Ethnicity	Saudi Arabian	NK	Northern European	Northern European	Syrian
Sex	M	NK	F	F	F
Age	5y	NK	Died 4y10	4y	7y
OFC (cm) [SD]	NK	Microcephaly	Primary microcephaly	46 cm (3 cm<br>below 3rd percentile)<br>Primary microcephaly	47cm
Development					
Developmental impairment	✓- global developmental delay	✓- global developmental delay with developmental regression	✓- profound, no cognitive and motor development	✓- profound	✓ - profound, no milestones
Neurology					
Findings	“Cerebral palsy”	Spasticity<br>abnormality of movement	progressive<br>spasticity	severe spasticity of all four limbs	Spasticity and contractures;<br>wheelchair bound,<br>little purposeful movement
Neurology - other					
Seizures	✓	✓	✓ - from 5m, infantile spasms then polymorphic and treat-ment resistant	✓ - from 2y electroencephalog-raphy<br>showed multifocal discharges	(✓) Febrile
Optic atrophy		NK	✓	✓ and abnormal visual evoked potentials (VEP).	Significant visual impairment
Neuroimaging					
MRI brain	Severe white matter volume loss with enlarged ventricles<br><br>Mild cerebellar atrophy<br>Thin corpus callosum<br>Cystic encephalomalacia	brain atrophy	Severe white matter volume loss with enlarged ventricles,<br>Absent myelination,<br>Hypoplastic corpus callosum	Severe white matter volume loss with enlarged ventricles,<br>Absent myelination,<br>Cerebellar atrophy<br>Thin corpus callosum	(reported by parents)<br>Brain atrophy and lissencephaly
Other					
	Gastroesophageal reflux dis-ease				

Table S2. Candidate *PTPN23* gene variants, proposed by Bend *et al.* 2020 [5].

	GRCH38 nomenclature NM_015466.4:	gnomAD v2.1.1 non-neuro (AF)	gnomAD v3.1 non-neuro (AF)	SiFT	Polyphen2 HumVar	Rationale for exclusion
Patient 1	Chr3:g.47410478C>T c.2680C>T p.(His894Tyr)	1 het (<0.01%)	4 het (<0.01%)	Tolerated	Benign	
	Chr3:g.47410545A>G c.2747A>G p.(Gln916Arg)	7 het (0.003%)	4 het (<0.01%)	Tolerated	Benign	-
Patient 2	Chr3:g.47411546G>A c.3748G>A p.(Glu1250Lys)	20 het (<0.01%)	18 het (0.01%)	Tolerated	Possibly damaging	-
	Chr3:g.47410663 CCAGCCCCATCCT>C c.2878_2889del12; p.(Gln960_Pro963del)	321 het (0.14%)	260 het (0.2%)	-	-	Poor amino acid conserva-tion / repeti-tive region (a)
Patient 3	Chr3:g.47408451C>T c.1291C>T p.(Arg431Trp)	36 het (0.01%)	25 het (0.01%)	Damaging	Possibly damaging	-
	Chr3:g.47410284C>T c.2486 C > T p.(Pro829Leu)	2 het (<0.01%)	3 het (<0.01%)	Damaging	Benign	-
Patient 4	Chr3:g.47410366 AGTT-GCAGGTCTCC CCTCGGCCCCACC > - c.2568_2594del27; p.(Val857_Pro865del)	Absent[b]	Absent	-	-	-
Patient 5	Chr3:g.47411681GAGA>G c.3884_3886delAGA; p.(1295_1296delLys)	113 het (0.05%)	50 het (0.03%)	-	-	**Ashkenazi Jewish gno-mAD v2.1.1 frequency 1.4%**[c]
Patient 6	Chr3:g.47412926C>CCC c.4651_4652dup; p.(Leu1552Hisfs*33)	Absent	Absent	-	-	-
Patient 7	Chr3:g.47409268A>G c.1748A>G p.(Lys583Arg) *in cis*	20 het (<0.01%)	50 het (0.03%)	Damaging	Possibly damaging	***in cis with*** **Gln1017His**
	Chr3:g.47410849G>C c.3051G>C p.(Gln1017His) *in cis*	59 het (0.03%)	49 het (0.04%)	Tolerated	Benign	***in cis with*** **Lys583Arg**
	Chr3:g.47406548G>A c.695 G > A p.(Arg232Gln) *in trans*	10 het (<0.01%)	14 het 1 hom (0.01%)	Damaging	Benign	**Homozygous individual in gnomAD 3.1.1**

Abbreviations: AF, allele frequency; het, heterozygous individual; hom, homozygous individual

(a) Note that this deletion can also be annotated as c.2866_2877del12 p.(Gln956_Pro959del) as it actually is a deletion of a once-repeated 12 nucleotide sequence (repeat QPHP), thus making the position of the change potentially ambiguous to mapping software. This region of the protein is poorly conserved (Fig S4) and although we can not conclude that the variant is benign we would consider it a variant of uncertain significance with limited evidence supporting causality.

(b) An alternative allele also resulting in the same p.Val857_Pro865del is present in one individual in gnomAD v2.1.1

(c) Assuming Mendelian ratios then it is expected that 1 in every 20,408 Ashkenazi Jewish individuals would be homozygous, suggesting the existence of about 600 such people

Table S3. Candidate *PTPN23* gene variants identified in patients with non-specific developmental impairment by Bend *et al.* 2020 [5] not excluded by allele frequency data in population databases

**Reference**	**Patient 1**[5]	**Patient 2**[5]	**Patient 3**[5]
Genotype (NM_015466.4)	c.2680C>T p.(His894Tyr) | c.2747A>G p.(Gln916Arg)	c.3748G>A p.(Glu1250Lys) | c.2878_2889del12; p.(Gln960_Pro963del)	c.1291C>T; p.(Arg431Trp) | c.2486C>T; p.(Pro829Leu)
Family history			
Ethnicity	Kuwaiti	European	North American
Sex	M	M	M
Age	14m	3y	6y
OFC (cm) [SD]	normal	normal	normal
Development			
Developmental impairment	✓ "Gross and fine motor skills at 12.5m corrected age were at 9m"	✓ "late to walk independently (~18 months)"	✓
Neurology			
Findings	nil	bilateral intention tremor and shuffling gait	Apraxia
Neurology - other			
Seizures	✗	✗ but abnormal EEG	✗
Optic atrophy	anomalous looking optic disc	NA	✗
Neuroimaging			
MRI brain	Ventriculomegaly with mild decrease in cerebral volume	Ventriculomegaly suspicious for brain volume loss	unremarkable
Other			
	Dysmorphic features, tracheoesophageal fistula, mild left pelviectasis	Dysmorphic features, Hypopigmented 4cm patch	VSD/PDA, autism, dyspraxia, hyperlexia, Sensory processing disorder

Supplementary Figure S4. Position of previously published *PTPN23* candidate missense variants and small in-frame deletions in relation to known PTPN23 protein domain architecture.

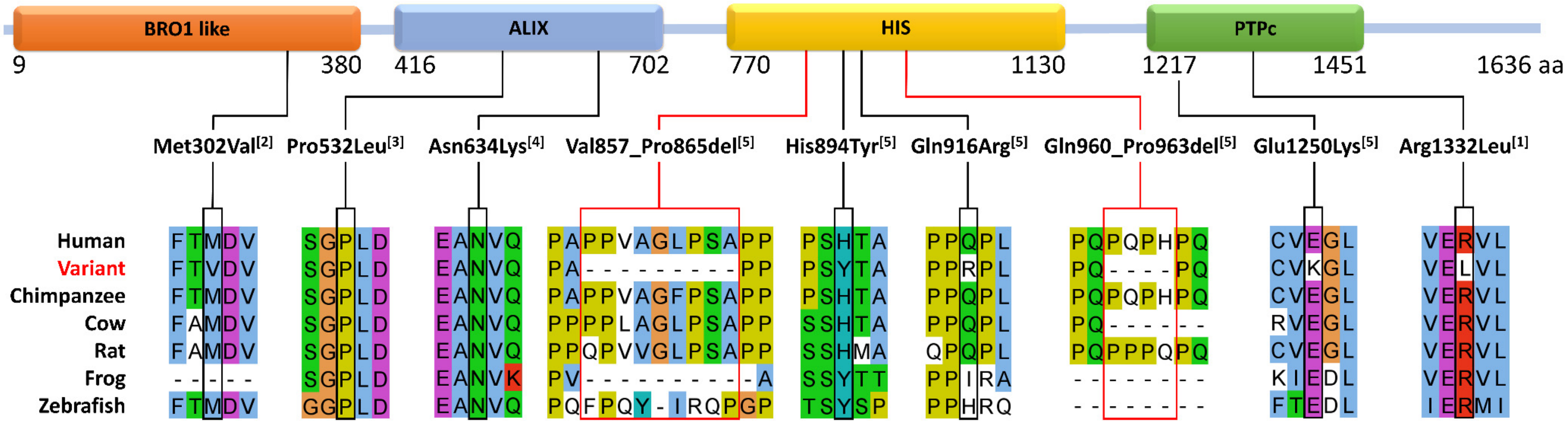

References for Supplemental paper

1. Alazami AM, Patel N, Shamseldin HE, Anazi S, Al-Dosari MS, Alzahrani F, et al. Accelerating novel candidate gene discovery in neurogenetic disorders via whole-exome sequencing of prescreened multiplex consanguineous families. Cell Rep. 2015;10(2):148-61. Epub 2015/01/06. doi: 10.1016/j.celrep.2014.12.015. PubMed PMID: 25558065.

2. Trujillano D, Bertoli-Avella AM, Kumar Kandaswamy K, Weiss ME, Köster J, Marais A, et al. Clinical exome sequencing: results from 2819 samples reflecting 1000 families. Eur J Hum Genet. 2017;25(2):176-82. Epub 2016/11/17. doi: 10.1038/ejhg.2016.146. PubMed PMID: 27848944; PubMed Central PMCID: PMCPMC5255946 employed at Centogene AG; AR has financial holdings in Centogene AG; WE, MTAR, AAR, WAT, AAlo, MAB, and MA are employees at King Abdulaziz Medical city; AAlh is employee at Prince Sultan Military Medical City; NAS is employee at Johns Hopkins Aramco hospital.

3. Sowada N, Hashem MO, Yilmaz R, Hamad M, Kakar N, Thiele H, et al. Mutations of PTPN23 in developmental and epileptic encephalopathy. Hum Genet. 2017;136(11-12):1455-61. Epub 2017/11/02. doi: 10.1007/s00439-017-1850-3. PubMed PMID: 29090338.

4. Smigiel R, Landsberg G, Schilling M, Rydzanicz M, Pollak A, Walczak A, et al. Developmental epileptic encephalopathy with hypomyelination and brain atrophy associated with PTPN23 variants affecting the assembly of UsnRNPs. European Journal of Human Genetics. 2018;26(10):1502-11. doi: 10.1038/s41431-018-0179-2.

5. Bend R, Cohen L, Carter MT, Lyons MJ, Niyazov D, Mikati MA, et al. Phenotype and mutation expansion of the PTPN23 associated disorder characterized by neurodevelopmental delay and structural brain abnormalities. European Journal of Human Genetics. 2020;28(1):76-87. doi: 10.1038/s41431-019-0487-1.
